# Diabetes personalized: the use of whole-exome sequencing in order to identify familial type 2 diabetes mellitus susceptibility factors

**DOI:** 10.1186/1471-2164-15-S2-P51

**Published:** 2014-04-02

**Authors:** Nehad Shaer, Jalaluddin J Khan, Ashraf Dallol, Adel Abuzenadah

**Affiliations:** 1 KACST Technology Innovation Center in Personalized Medicine, King Abdulaziz University, Jeddah, KSA; 2 Department of Biochemistry, Faculty of Science, King Abdulaziz University, Jeddah, KSA

## Background

Type 2 Diabetes Mellitus (T2DM) is a complex and pleomorphic metabolic disorder arising from a complex interaction between genes and the environment. However, the molecular landscape of T2DM is not fully explored, especially in a highly consanguineous society as the Saudi Arabian population [1]. Extended families could be predicted to cause an increase in the number and severity of genetic susceptibility factors for T2DM. We explore this hypothesis by applying whole-exome sequencing on four members of a Saudi family who all suffer from T2DM.

## Materials and methods

Whole-exome sequencing of genomic DNA extracted from peripheral blood is performed on 3 brothers and their mother who are all suffering from confirmed T2DM. Sequencing was performed on the SOLiD 5500 XL platform at the Center of Excellence in Genomic Medicine Research, Jeddah, Saudi Arabia.

## Results

We have identified novel mutations affecting SPRY2, ALPK2, ANXA4, and AGBL2 genes that could affect this family particularly susceptible to T2DM.

## Conclusions

Whole-exome sequencing is very useful tool for detecting a large fraction of mutations, however, it cannot determine other genetic aberrations such as copy number and structural variations. However, finding novel mutations in a T2DM affecting genes such as SPRY2 may implicate these genes in diabetes.
